# Evaluation of the efficacy of stomatitis prevention in prosthetics with complete dentures with additional fixation with the cream

**DOI:** 10.4317/jced.60141

**Published:** 2023-02-01

**Authors:** Vitaliy Shuturminskiy, Ihor Seredunko, Andrii Bas

**Affiliations:** 1 Odesa Medical Institute, International Humanitarian University. 65009, 33 Fontanska Road, Odesa, Ukraine

## Abstract

**Background:**

The objective of the study is to evaluate a microbiological efficacy of prevention and development of prosthetic stomatitis in full removable prosthetics.

**Material and Methods:**

All patients with complete absence of the teeth on the lower jaw were divided into four groups: individuals who used full removable dentures and did not use any fixation agents and adhered to conventional oral hygiene; patients with full removable dental prostheses who used the cream “Corega” to strengthen the fixation from the first day of prosthetics and adhered to conventional oral hygiene; patients with complete removable dentures who used Corega Comfort (GSK) to strengthen the fixation from the first day of prosthetics and adhered to conventional oral hygiene; patients with complete removable dentures who used Corega Comfort (GSK) to enhance the fixation and performed antibacterial cleaning of dentures using Biotablets “Corega” for cleaning dentures from the first day of prosthetics. Microbiological and mycological examination of the patients included microscopic examination of smears using conventional and luminescent methods of staining smears from the denture surface.

**Results:**

The data obtained show that probiotic species of microbial flora of the oral cavity are prone to colonization on the surface of complete removable acrylic dental prostheses when using fixation creams “Corega” and Corega Comfort (GSK), which is not characteristic of acrylic dentures without additional fixation. This flora much exceeds virulent one and Candida fungi quantitatively.

**Conclusions:**

It can be concluded that the use of complete removable dentures with the application of biotablets “Corega”, can significantly (one hundred times) reduce the contamination of the dental prosthesis after 1 month of the follow-up. In general, pathogenic inoculation the application of this type of denture hygiene the makes it possible to achieve reduction of the number of streptococcal colonies by several times.

** Key words:**Fixation gel, Candida fungi, patient, oral cavity, microbial content.

## Introduction

According to various authors, the prevalence of prosthetic stomatitis, one of the most common complications of complete removable prosthetics is from 47 to 60% of people who have had dentures (1-3). It should be noted that over time, when using them this percentage increases (4-6). Traumatic prosthetic stomatitis is one of the most common etiological forms, caused by injury to the prosthetic bed by the base of a removable plate denture. Microbiological and fungal factors are also related to the occurrence of prosthetic stomatitis (7). Complete removable prosthetics is a temporary or permanent alternative to prosthetics on dental implants (8), so the problem of improving the quality of this type of prosthetics and indirectly the quality of life of patients is not only a medical but also a social problem.

Unfortunately, the period of using complete removable dentures is limited to three years, and with each subsequent fabrication of the denture, the orthopedist faces the problem of deterioration of the anatomical and functional conditions of the prosthetic bed (9-11). Quite often the dentist has to resort to additional methods to improve the fixation and stabilization of a complete removable denture, especially when it is made on the edentulous lower jaw (12). In any case, however, patients, improve the fixation of a complete removable denture with a fixation gel or cream upon the recommendation of an orthopedic dentist or on their own initiative (13-15). Taking into account the prevalence of fixation creams in full removable prosthetics and the prevalence of prosthetic stomatitis as a complication of this type of prosthetics, author set the aim of the study – to conduct a microbiological evaluation of the efficacy of prevention and development of prosthetic stomatitis in full removable prosthetics.

## Material and Methods

The study involved patients aged 50 to 59 years who were made complete removable dentures for the lower jaw for the first time. The distribution of the patients by gender, the degree of atrophy of the alveolar process and the condition of the mucous membrane are presented in Table 1.

**Table 1:**
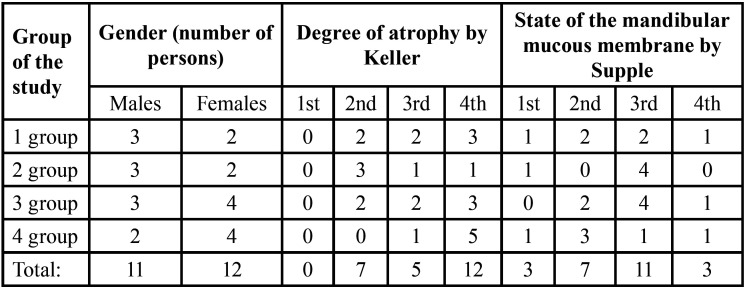
Distribution of patients by gender and anatomical and physiological conditions of the prosthetic bed.

All patients – 23 persons in total were divided into four groups:

- group 1 – persons who used full removable dentures and did not use any fixations and adhered to conventional oral hygiene;

- group 2 – patients with full removable dentures who used the cream “Corega” to strengthen the fixation from the first day of prosthetics and adhered to conventional oral hygiene;

- group 3 – patients with complete removable dentures who used Corega Comfort (GSK – GlaxoSmithKline) to strengthen the fixation from the first day of prosthetics and adhered to conventional oral hygiene;

- group 4 – patients with complete removable dentures who used Corega Comfort (GSK) and performed antibacterial cleaning of dentures with Biotablets “Corega” for cleaning dentures to enhance the fixation from the first day of prosthetics.

The dentures were made of acrylic resin and clinical methods of additional fixation were used if necessary. Mycological examination of the patients included microscopic examination of smears using conventional and luminescent methods of staining smears from the surface of the dentures. Cultural studies with quantitative estimation of the fungi were also conducted. The patients were carried out studies after prosthetics in the terms: 14 days, 1, 2, 6 months after denture fitting. Bacteriological investigations were conducted to study the number and biological properties of microorganisms on the surface of the dentures. A sterile cotton swab soaked in saline was used to take the material from the surface of the dentures for isolation and quantitative estimation of the microorganisms followed by inoculation of the material on yolk-salt agar (YSA). The selection of test colonies on YSA was made after incubation at a temperature of 37°C for 2 days.

The basis for the quantitative estimation of colonies was the severity of the lecithinase reaction (formation of a cloudy zone and a rainbow crown around the colony). Microscopic methods were used to determine the species of selected fungal cultures. To quantify the selected cultures of the fungi, the material was taken from the surface of the denture on an empty stomach. A sterile cotton swab, turning round, carefully wiped the surface of the denture. Then the swab was immersed in a vial of 5 ml of saline and sterile balls. The contents of the vial have been shaken for 5 minutes. Then 0.1 ml of the liquid from the vial was inoculated into a Petri dish with Saburo agar. To suppress other microbial flora and obtain a pure culture of fungi penicillin and streptomycin was added to the culture medium at the rate of 100 IU (international unit) per 1 ml of the medium. The material was evenly distributed on the agar surface with a Drygalsky spatula. The cultures have been incubated at 37°C for 48 hours. To quantify the results, cultures in Petri dishes were divided into a number of sectors, where the number of grown colonies was counted. Then the total number of colonies was determined and multiplied by the degree of dilution of the pathological material in saline.

## Results

After fitting acrylic complete removable denture, the number of colonies of streptococci reached the level (1.24 ± 0.58) 107 col./cm2 on the 14th day of using (Table 2).

**Table 2:**
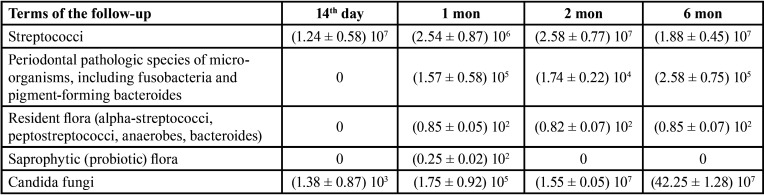
The results of the study of microbial colonization of the oral cavity and removable dentures in the patients of the 1st group, M ± m, col./cm2 in dynamics.

**Figure 1:**
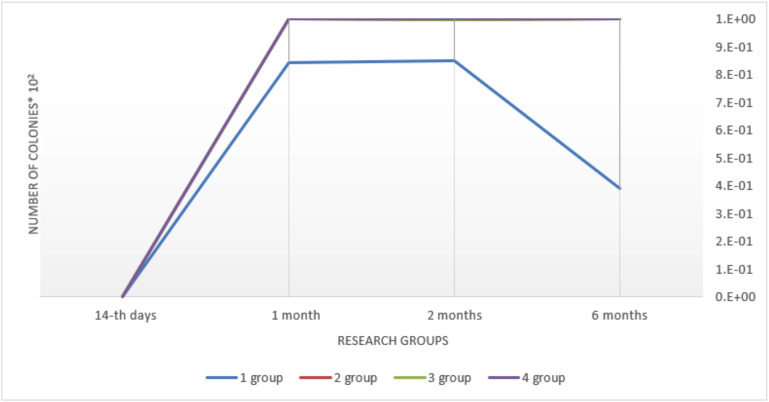
Comparative characteristics of insemination of removable dentures of Periodontal pathologic species of microorganisms when used different fixing gels (col./sm * 102).

While studying colonization of dentures by periodontopathogenic microorganisms, author observed their main colonization in 1 month – (1.757 ± 0.58) 105 col./cm2, which stabilized and remained at a fairly high level for up to 6 months in this group – (2.58 ± 0.75) 105 col./cm2 (Fig. 1).

**Figure 2:**
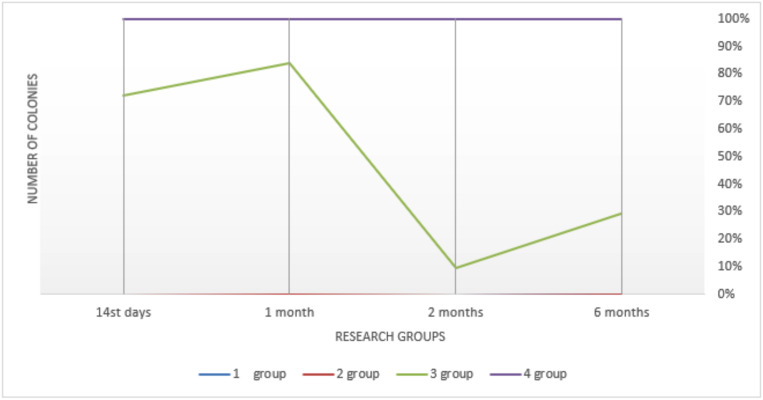
Comparative characteristics of insemination of removable dentures with resident flora when used different fixing gels (col./sm).

As for the resident flora that can support the course of purulent inflammation due to a sharp increase in the amount of toxins (α-greenish streptococci, peptostreptococci and bacteroides), their colonization was insignificant in this group, was found only on the 30th day (0.85 ± 0.05) 102 and remained at this level until the 6th month of the study (Fig. 2).

However, it is noteworthy that the saprophytic (probiotic) flora in this group of studies was almost absent or manifested in small quantities on the 30th day. In the 2nd group of studies (Table 3) (the patients with complete removable dentures who used the fixation gel “Corega”) after 14 days of wearing colonies of streptococci on the surface of the base made (5.45 ± 0.44) 107 col./cm2, which significantly exceeded the indices of the 1st group.

**Table 3:**
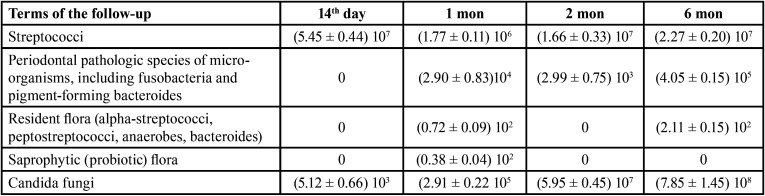
The results of the study of microbial colonization of the oral cavity and removable dentures in the patients of the 1st group, M ± m, col./cm2 in dynamics.

**Figure 3:**
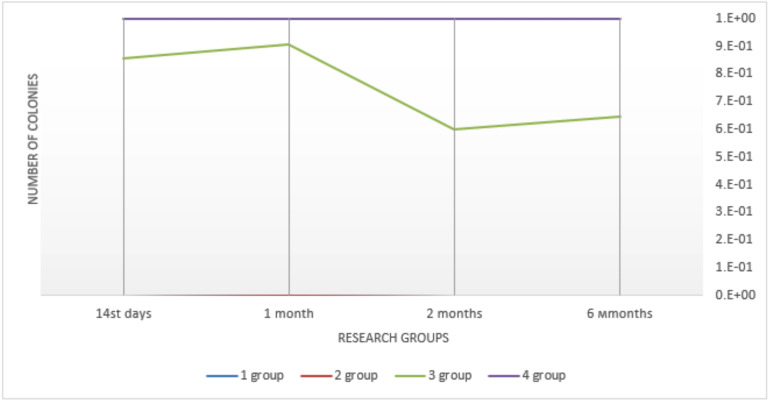
Comparative characteristics of insemination of removable dentures with probiotic flora when used different fixing gels (col./sm * 102).

In 1 month, the number of streptococci decreased and reached the level of (1.77 ± 0.11) 106 col./cm2 and remained at this level without significant change throughout the study (Table 3). Colonization by resident flora was similar, which was absent on the 14th day. Colonization peak occurred in 6 months – (2.11 ± 0.15) 102 col./cm2. High rates of contamination by the main pathogenic flora of the dentures in the oral cavity in group 2 prove that fixation gels increase the deposition of the pathogenic flora on the denture surface due to the fatty structure and their colonization on the mucosa increases, which is not available for self-cleaning (Fig. 2). The absence or inconsistency of saprophytic (probiotic) flora in both representative groups indicate, in turn, the displacement of this important group of microorganisms due to the excessive development of α-streptococci, peptostreptococci and virulent anaerobic bacteria (Fig. 3).

As for *Candida fungi*, they were determined almost through the follow-up period in the 3rd group although in small quantities: from (0.02 ± 0.005) 102 col./cm2 on the 14th day to (2.42 ± 0.42) 102 col./cm2 – in 1 month (Table 4).

**Table 4:**
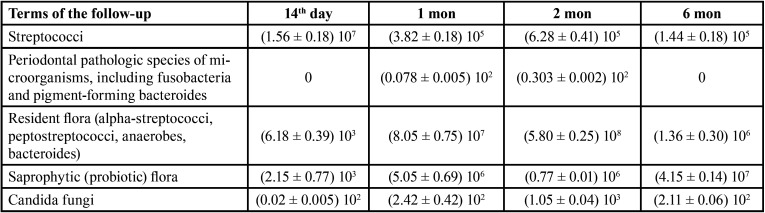
The results of the study of microbial colonization of the oral cavity and removable dentures in the patients of the 3rd group, M ± m, col./cm2 in dynamics.

The results of the study in the 4th group (in the patients with complete removable dentures when using a fixation cream and bioTablets for cleaning “Corega”) are presented in Table 5.

**Table 5:**
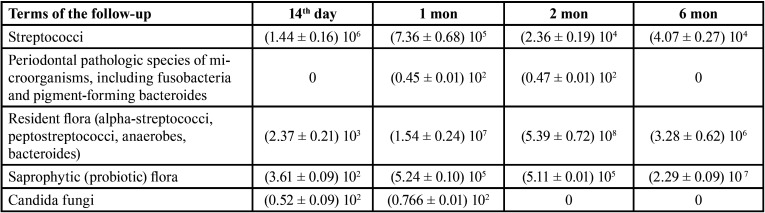
The results of the study of microbial colonization of the oral cavity and removable dentures in the patients of the 4th group, M ± m, col./cm2 in dynamics.

**Figure 4:**
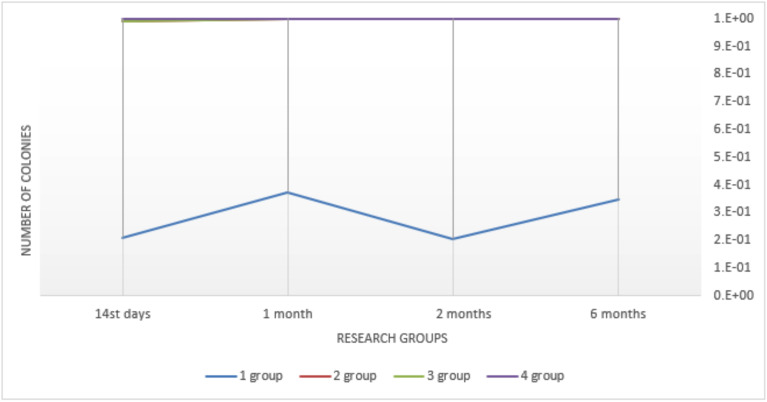
Comparative characteristics of insemination of removable dentures of Candida fungi when used different fixing gels (col./sm2 * 102).

On the 14th day after fitting a removable denture, the number of streptococcal colonies on the surface of the base was (1.44 ± 0.16) 106 col./cm2, which was the lowest index of all study groups. By 6 months streptococcal colonization decreased to the lowest level – 4.07 ± 0.27 104 col./cm2. Colonization by the saprophytic flora was quite active, reaching a maximum in 2 months – (5.11 ± 0.01) 105 col./cm2, which was also the best result (Fig. 4).

## Discussion

According to I.S. Albuquerque *et al*. (12), plaque on prostheses and their insufficient cleaning are sometimes accompanied by prosthetic stomatitis. The most common cause of prosthetic stomatitis is *Candida albicans*. As a result of insufficient hygiene on the part of the user of the removable prosthesis, who are more often people of older age groups, a thin, stable protein coating is deposited on its surface, on which food particles settle, creating a breeding ground for bacteria. R.S. Menon *et al*. (13) believe that bacteria actively multiply, forming calcifications on the surface of the removable prosthesis, which become pigmented if they are not removed in time from the surface of the prosthesis. According to M. Gul *et al*. (14), the toxic effect of various types of prostheses on the mucous membrane enhances the adhesion of pathogenic microorganisms. L. Lepidi *et al*. (15) note that more than thirty bacterial species are considered residents of the oral cavity, but due to changes in the microbiocenosis of the oral cavity, they can lead to the development of the disease.

The data obtained show that probiotic species of the microbial flora of the oral cavity are prone to colonization on the surface of complete removable acrylic dental prostheses when using fixation creams “Corega” and Corega Comfort (GSK), which is not typical of acrylic dentures without additional fixation. This flora much exceeds virulent one and *Candida fungi* quantitatively (16). Regarding the contamination by *Candida fungi*, it should be noted that the increase in the number of fungal colonies occurred in arithmetic progression and reached the level (42.25 ± 1.28) 107 col./cm2 on the 6th month of the study that was often accompanied by certain symptoms in the clinical course (dry mouth, burning in the denture area, etc.).

It can also be stated that usage of complete removable dentures with the application of biotablets “Corega” allows significantly (one hundred times) reducing the contamination of the dental prosthesis after 1 month of the follow-up. In general, pathogenic contamination the application of this type of denture hygiene makes it possible to achieve reduction in the number of streptococcal colonies by several times (17-18).

## Conclusions

The results obtained allow us to state that when applying the fixation cream “Corega” in combination with biotablets “Corega” it is possible to observe a more favorable microbial impact on the oral cavity microbiocenosis due to the above-mentioned properties. Based on the data obtained, the risk of prosthetic stomatitis of mitotic or bacterial etiology is much higher in people who use full removable acrylic dentures without fixation cream and with conventional oral hygiene. The use of creams slightly increases the streptococcal colonization on the surface of dental prostheses, and over time the saprophytic flora neutralizes the growth of streptococcal colonies. When using prosthetic hygiene with the use of biotablets “Corega” it has been the best result of disinfecting impact on the surface of the denture.
